# Hyperkinetic Movement Disorder Caused by the Recurrent c.892C>T *NACC1* Variant

**DOI:** 10.1002/mdc3.14051

**Published:** 2024-05-02

**Authors:** Jonna Komulainen‐Ebrahim, Salla M. Kangas, Estrella López‐Martín, Timothy Feyma, Fernando Scaglia, Beatriz Martínez‐Delgado, Outi Kuismin, Maria Suo‐Palosaari, Lucinda Carr, Reetta Hinttala, Manju A. Kurian, Johanna Uusimaa

**Affiliations:** ^1^ Research Unit of Clinical Medicine University of Oulu Oulu Finland; ^2^ Medical Research Center Oulu University Hospital, University of Oulu Oulu Finland; ^3^ Department of Children and Adolescents, Division of Pediatric Neurology Oulu University Hospital Oulu Finland; ^4^ Biocenter Oulu, University of Oulu Oulu Finland; ^5^ Institute of Rare Diseases Research, Instituto de Salud Carlos III Madrid Spain; ^6^ Gillette Children's Specialty Healthcare Saint Paul Minnesota USA; ^7^ Department of Molecular and Human Genetics Baylor College of Medicine Houston Texas USA; ^8^ Texas Children's Hospital Houston Texas USA; ^9^ Joint BCM‐CUHK Center of Medical Genetics, Prince of Wales Hospital Shatin Hong Kong; ^10^ Department of Clinical Genetics Oulu University Hospital Oulu Finland; ^11^ Department of Diagnostic Radiology Oulu University Hospital Oulu Finland; ^12^ Research Unit of Health Sciences and Technology University of Oulu Oulu Finland; ^13^ Department of Neurology Great Ormond Street Hospital London United Kingdom; ^14^ Developmental Neurosciences, Zayed Centre for Research into Rare Disease in Children UCL Great Ormond Street Institute of Child Health London United Kingdom

**Keywords:** cyclic, hyperkinetic, movement disorder, NACC1

## Abstract

**Background:**

Genetic syndromes of hyperkinetic movement disorders associated with epileptic encephalopathy and intellectual disability are becoming increasingly recognized. Recently, a de novo heterozygous *NACC1 (*nucleus accumbens‐associated 1) missense variant was described in a patient cohort including one patient with a combined mitochondrial oxidative phosphorylation (OXPHOS) deficiency.

**Objectives:**

The objective is to characterize the movement disorder in affected patients with the recurrent c.892C>T *NACC1* variant and study the NACC1 protein and mitochondrial function at the cellular level.

**Methods:**

The movement disorder was analyzed on four patients with the *NACC1* c.892C>T (p.Arg298Trp) variant. Studies on NACC1 protein and mitochondrial function were performed on patient‐derived fibroblasts.

**Results:**

All patients had a generalized hyperkinetic movement disorder with chorea and dystonia, which occurred cyclically and during sleep. Complex I was found altered, whereas the other OXPHOS enzymes and the mitochondria network seemed intact in one patient.

**Conclusions:**

The movement disorder is a prominent feature of NACC1‐related disease.

Childhood movement disorders comprise a heterogeneous group of disorders that lead to the impairment of voluntary movements, abnormal postures, or inserted involuntary movements.^1^ Movement disorders in children are often classified into two main categories: hyperkinetic/dyskinetic (including stereotypies, tics, tremor, dystonia, chorea, athetosis, and myoclonus) and hypokinetic (encompassing parkinsonian phenotypes).^1^, ^2^ Hyperkinetic movement disorders are commonly attributed to dysfunction of the basal ganglia, cerebral cortex, cerebellum, and other motor pathways because of static or progressive injury.^1^

Underlying etiologies are diverse, including both acquired and genetic conditions.^2^ The symptoms commonly overlap with the clinical features observed in mitochondrial disorders and other neurogenetic diseases—for example, glucose transporter type 1 deficiency syndrome (GLUT1DS).^3^, ^4^

Advances in molecular genetics have discovered various novel genes responsible for pediatric movement disorders as part of neurodevelopmental diseases. There are several genetically and clinically heterogeneous disorders that involve both hyperkinetic movement disorder and epileptic encephalopathy and usually present in combination with developmental disability. Several genes associated with rare disorders can be responsible for these phenotypes. In addition, variants in a given gene can be associated with several phenotypes, which are often part of a spectrum and not discrete entities.^5^ In combined pediatric and adult patient populations, the diagnostic yield of next‐generation sequencing (NGS) panels and whole exome sequencing (WES) is estimated at between 14.8% and 20%;^6^, ^7^ however, in pediatric cohorts, the yield is usually higher—32% to 51%.^4^, ^8^

Recently, a de novo heterozygous *NACC1* (nucleus accumbens‐associated 1), HUGO Gene Nomenclature Committee (HGNC) Identifier HGNC:20967, c.892C>T (NM_052876.4; NP_443108.1: p.Arg298Trp) variant has been described in nine patients with infantile onset epilepsy, postnatal microcephaly, severe to profound intellectual disability, bilateral cataracts, and hyperkinetic movements including hand stereotypies, chorea, and dystonia.^9^, ^10^, ^11^ Furthermore, one of these patients also exhibited combined oxidative phosphorylation (OXPHOS) deficiency.^9^ *NACC1* encodes nucleus accumbens‐associated protein 1 (NACC1), which is also known as BTB/POZ domain‐containing protein 14B (BTBD14B), and it is a multifunctional protein that has been shown to act as a versatile transcription factor, but it also plays a role in protein turnover.^12^, ^13^

In this study, we describe a patient cohort of four patients to illustrate the hyperkinetic movement disorder associated with the recurrent missense *NACC1* variant. We also report treatment responses for the currently available drugs. Patient‐derived fibroblasts were used to study the effects of this variant on NACC1 expression, localization, and mitochondrial function.

## Materials and Methods

### Clinical Features and Genetic Testing

Clinical information was collected from the medical reports and the parents of four patients harboring the recurrent de novo heterozygous *NACC1* missense c.892C>T (p.Arg298Trp) (NM_052876.4) variant found by WES. Patient 3 in this study was previously described as participant 5 by Schoch et al.^9^ Analysis of movement semiology was undertaken by M.A.K. and L.C. (Great Ormond Street Hospital) using the videos of the patients, recorded at different ages (patient 1 from 1 year to 3 years of age, patient 2 from 1 year to 6 years of age, patient 3 from 1 year to 12 years of age, and patient 4 at 14 years of age). The videos were reviewed independently, with consensus agreement for any noted differences.

### Functional Studies on Patient‐Derived Fibroblasts

To study the effect of the *NACC1* p.Arg298Trp variant on cellular and mitochondrial function in vitro, cultured fibroblasts were obtained from one patient (patient 1). Commercially available fibroblast cell lines derived from healthy adults were used as controls. Methods for cell culture, reverse transcription polymerase chain reaction (RT‐PCR), quantitative RT‐PCR, immunocytochemistry, western blotting, Blue Native (BN) polyacrylamide gel electrophoresis (PAGE) and in‐gel activity assay are described in detail in Data S1.

## Results

### Clinical Features of the Movement Disorder

All four patients presented with a complex neurological phenotype, including cyclic dysautonomia, extreme irritability, and insomnia; profound intellectual disability; postnatal microcephaly; epilepsy; bilateral cataracts; iron deficiency anemia; and feeding difficulties leading to the requirement of tube feedings. Clinical features, detailed descriptions of each patient's movement disorder, and movement analyses are presented in detail in Data S2.

The movement disorder was recognized between 2 months and 1 year of age. Generalized hyperkinetic movement disorder included myoclonus, dystonia, chorea, orolingual dyskinesia, and sleep‐related paroxysmal dyskinesias in all four patients. Spasticity was present in three patients and increased with age in two patients. The fourth patient also experienced muscle hypertonia during insomnia periods. All four patients experienced sleep‐related paroxysmal dyskinesias. The hyperkinetic movements and spasticity or muscle hypertonia were more prominent during irritability and insomnia periods in all the patients. Stereotypic hand clasping in midline, hand mouthing, and biting began by the time the children were 3 to 5 years old. Several drugs were trialed, and mild to moderate effects was seen with clonidine in three patients, moderate effect with onabotulinum toxinA and tetrahydrocannabinol in one patient, and moderate effect with clonazepam in one patient. Baclofen had a mild effect on muscle hypertonia in one patient.

The characteristics of the patients' movement disorder and clinical features are summarized in Table 1. The videos reveal the features of the movement disorder for patient 1 (Video 1), patient 2 (Video 2), and patient 4 (Video 3).

**TABLE 1:** Movement disorder characteristics and main clinical features associated with pathogenic heterozygous *NACC1* c.892C>T (p.Arg298Trp)

	Patient 1	Patient 2	Patient 3^9^	Patient 4
Age, y	6	9	15	16
Gender	Male	Male	Female	Male
*NACC1* c.892C>T (p.Arg298Trp) de novo variant	Yes	Yes	Yes	Yes
Age of onset (movement disorder)	6 months	9 months	1 y	2 months
Myoclonus	+	+	+	+
Dystonia	+	+	+	+
Dystonic crises^a^	−	+	−	−
Chorea	+	+	+	+
Orolingual dyskinesia	+	+	+	+
Stereotypic behavior	+	+	+	+
Sleep related dyskinesias	+	+	+	+
Spasticity	−	+	+	+
Evolution of movement disorder	+^b^	−	+^c^	+^d^
Drugs trialed for movement disorder and effect	CLO+^e^ CLZ++^f^ GBP− NTZ− DZP− LZP− OZP− BLF+^g^	CLO ++^h^ DZP−	LZP− DZP−	CLO+^i^ TCH++^j^ BTX++^j^ GBP− CPH− CH− BLF− CLZ− DZP− GFC−
Cyclic dysautonomia, irritability, and insomnia	+	+	+	+
Epilepsy	+	+ IS	+ IS	+ IS
Profound intellectual disability	+	+	+	+
Bilateral cataracts	+	+	+	+
GI problems/feeding difficulties	+	+	+	+
Iron‐deficiency anemia	+	+	+	+
Microcephaly	+	+	+	+
Mitochondrial dysfunction	(+)^k^	NA	+^l^	+^m^
Brain MRI	Delayed myelination, thin corpus callosum, mild decrease in brain volume	Normal	Delayed myelination, minimal volume loss	Delayed myelination, diffuse atrophy

Abbreviations: *NACC1*, nucleus accumbens‐associated 1; +, mild effect, −, no effect; CLO, clonidine; LZP, lorazepam; CLZ, clonazepam; ++, moderate effect; DZP, diazepam; TCH, tetrahydrocannabinol; GBP, gabapentine; BTX, onabotulinum toxinA; NTZ, nitrazepam; CPH, cyproheptadine; CH, chloral hydrate; OZP, oxazepam; BLF, baclofen; GFC, guanfacine; IS, infantile spasms; GI, gastrointestinal; NA, not assessed; MRI, magnetic resonance imaging.

^a^ Occasionally requiring hospitalization.

^b^ Stereotypic hand mouthing and biting during hyperactive stage increased at the age of 5 y.

^c^ Stereotypical hand clasping in midline, hand mouthing, and biting was present by 3 y of age. Intensity of the hyperkinetic movements during irritability and insomnia periods have reduced with age.

^d^ Stereotypical hand clasping and hand mouthing were observed by 3 y of age. Spasticity has increased with age.

^e^ Has been partially helpful for insomnia, vomiting, and tachycardia.

^f^ Has usually helped with the hyperkinetic movement disorder and partially with muscle hypertonia.

^g^ Partially helpful for muscle hypertonia.

^h^ Has been effective for dystonic crises and spasticity but has not had an effect on choreic tremulous jerks during sleep.

^i^ Has been helpful in promoting rest.

^j^ Has been helpful for spasticity and involuntary movements.

^k^ Complex I activity, when normalized to the level of the fully assembled enzyme complex, was detected as being decreased in patient‐derived fibroblasts.

^l^ Muscle biopsy showed a reduction in several respiratory chain complexes, including complexes I and IV6.

^m^ Citrate synthase activity was increased, thereby suggesting mitochondrial proliferation, and the activities of several respiratory chain complexes were reduced fulfilling minor modified Walker criteria, with a more severe deficiency of complex I activity.

**Video 1:** Patient 1 with cyclic hyperkinetic movement disorder and sleep‐related paroxysmal dyskinesia. Segment 1: Age, 2 years and 4 months. He is lying supine on the mat. There is limited social engagement with the adults around him or with the toy he is given. Hyperkinetic movement disorder with jerky, dyskinetic, and occasionally athetoid movements of the upper limbs and repeated episodes of tongue protrusion and mouthing, facial grimacing, and limb posturing suggestive of dystonia (fisted hands striatal toe, toe clawing). Marked axial hypotonia and head lag when pulling to sit and when sitting. Segment 2: Age, 3 years and 6 months. More prominent generalized hyperkinetic jerky movement disorder with orolingual dyskinesia, upper and lower limb posturing (fisted hands), and striatal toe/foot clawing from time to time. Segment 3: Age, 3 years. During sleep, there is episodic generalized jerky and low to moderate amplitude choreiform movements with a few possibly tremulous and jerky movements (possible myoclonus) predominantly of the upper limbs, but also affecting the lower limbs. The episodes are short and appear to last for <30 seconds. At times, there is either fisting or dystonic posturing of the right hand. Perioral dyskinesia is also present.

**Video 2:** Patient 2 with hyperkinetic movement order and sleep‐related paroxysmal dyskinesia. Segment 1: Age, 4 years and 6 months. An opisthotonic posture, with retrocollic neck posture, truncal arching, and extension of the legs and bilateral equinus posturing of the feet. The right arm is repeatedly lifted and held in dystonic posture, arm extended, wrist flexion, and in pronation on one occasion. His legs are intermittently flexed. Segment 2: Age, 6 years and 6 months. There is tongue protrusion and a tendency to bring his hands to his mouth. There are some low amplitude stereotyped movements of the hands in the midline, with a degree of hand fisting. From time to time, there is elevation of both legs and possibly similar, rather subtle choreiform movements distally in the lower limbs. Some paroxysmal eye blinking and oromotor movements are also noted. Segment 3: Age, 6 years and 6 months. The child is lying on the bed. On turning, a few choreiform movements are evident, mostly in the hands and feet. Toe clawing is seen. Paroxysmal opening and closing of the mouth is also evident. Segment 4: Age, 6 years and 6 months. From sleep, there are episodic generalized jerky movements that are either choreiform or possibly myoclonic; these are evident on turning.

**Video 3:** Patient 4 with hyperkinetic movement order. Segment 1: Age, 14 years. Posture is flexed, except on one occasion when he throws his head back into an extensor posture. Upper limb voluntary movements appear jerky, dyskinetic, and occasionally athetoid. Some subtle hand and finger posturing is also noted. Elbows and knees are flexed at all times. Both feet show clawing postures. There is also intermittent jaw opening, tongue protrusion, and orolingual dyskinesia. He does not appear to be able to visually track an object. Segment 2: Age, 14 years. Upper limb dyskinesia while rolling from back to front. He is able to raise his head and trunk onto flexed arms. Elbows and knees are flexed. There is orolingual dyskinesia and repeated jaw opening with intermittent tongue protrusion. Foot clawing is also evident.

### NACC1 Expression and Mitochondrial OXPHOS Assembly Studies in Patient‐Derived Fibroblasts

**FIG. 1:**
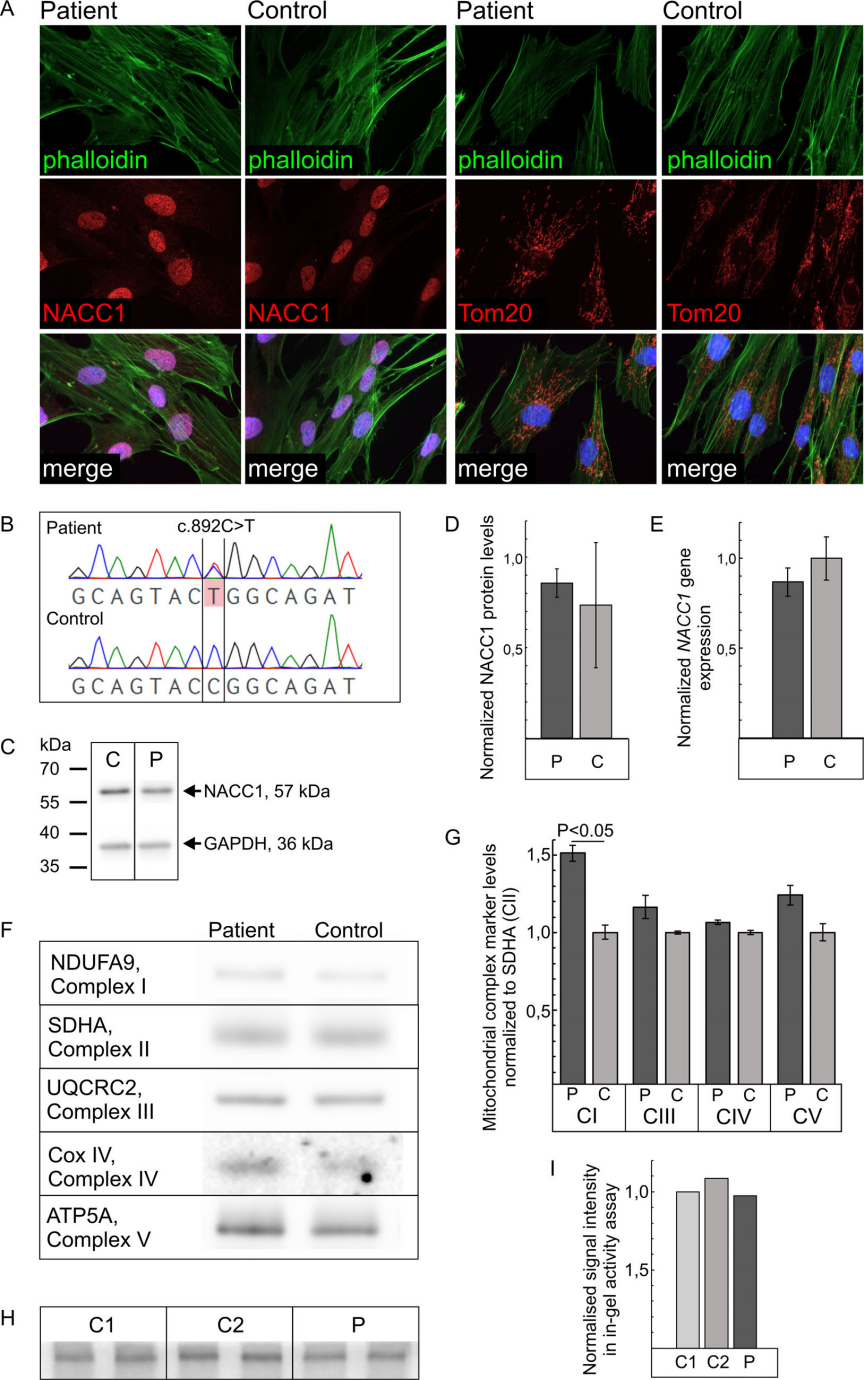
Cellular phenotype, nucleus accumbens‐associated protein 1 (NACC1) expression and expression, function, and assembly of mitochondrial complexes were studied in patient‐derived fibroblasts. (**A**) Patient‐derived fibroblasts from patient 1 have normal cell morphology and NACC1 is localized in the nucleus; a faint signal is observed in the cytoplasm both in patient‐derived cells and controls. Tom20 antibody was used to visualize the mitochondrial network, which appears normal in patient‐derived cells. Phalloidin:FITC was used to visualize actin cytoskeleton. Images were taken using 63× magnification. (**B**) Heterozygous expression of nucleus accumbens‐associated 1 (*NACC1*) c.892C>T variant in patient‐derived fibroblasts was verified using reverse transcription polymerase chain reaction (RT‐PCR) and Sanger sequencing. (**C**) Immunoblotting of cell lysates from patient‐derived (P) and control (C) fibroblasts with NACC1‐specific antibody. GAPDH was used as loading control. (**D**) NACC1 levels normalized to GAPDH revealed normal amount of 57‐kDa band detected by NACC1 antibody in patient fibroblasts (P) compared to controls (C). (**E**) *NACC1* gene expression level in patient‐derived cells was studied using quantitative PCR and it did not differ from the control cells. (**F**,**G**) Blue Native polyacrylamide gel electrophoresis and protein quantification derived from the western blot method indicated that complex I level in patient cells was 1.5‐fold compared to the control fibroblasts (*P* < 0.05, two‐tailed Student's *t* test). Other mitochondrial complexes were expressed at a normal level in patient‐derived fibroblasts. The levels of the marker proteins representative of mitochondrial complexes encoded by mitochondrial DNA were normalized to succinate dehydrogenase complex flavoprotein subunit A (SDHA), thereby representing complex II, which is encoded by the nuclear genome. (**H**,**I**) In‐gel activity assay to measure complex I function showed normal function in patient‐derived fibroblasts when compared to two control cell lines. Signal intensity was normalized to control 1 (C1) fibroblast line. Error bars in the images indicate standard deviation.

The de novo *NACC1* c.892C>T variant of the primary patient‐derived fibroblasts was confirmed using Sanger sequencing, and Sanger sequencing of the complementary DNA was used to show that the *NACC1* c.892C>T variant is expressed at the mRNA level (Fig. 1B). Further, to study the effect of the p.Arg298Trp variant on *NACC1* gene and protein expression levels, the patient‐derived fibroblasts were analyzed using immunoblotting and quantitative RT‐PCR (Fig. 1C–E). The *NACC1* transcript or protein levels showed no statistically significant change in its intensity in patient‐derived cells compared to the control. Next, the subcellular localization of NACC1 was studied using immunocytochemistry. The majority of the NACC1 signal was found to be normally localized into the nucleus, and the mitochondrial network was intact (Fig. 1A).

Before the genetic diagnosis of *NACC1* c.892C>T (p.Arg298Trp), two patients (patients 3 and 4) were suspected of having primary mitochondrial disease and underwent a muscle biopsy. Mitochondrial respiratory chain enzymatic activities in skeletal muscle of patient 3 showed a reduction in several respiratory chain complexes, including complexes I and IV.^9^ Citrate synthase activity was increased in patient 4, thereby suggesting mitochondrial proliferation, and the activities of several respiratory chain complexes were reduced fulfilling minor modified Walker criteria, with a more severe deficiency of complex I activity.

We conducted a detailed study of the levels of mitochondrial OXPHOS complexes in cultured fibroblasts from patient 1 using BN PAGE (Fig. 1F,G). Fully assembled complex I level was increased 1.5‐fold (*P* < 0.05) in the patient‐derived cells when compared to the control cell line. Interestingly, the in‐gel activity was similar to the control, thereby suggesting that complex I activity—when normalized to the level of fully assembled complex I—is decreased in the patient‐derived fibroblasts (Fig. 1H,I). The expression of other OXPHOS complexes was found to be normal in patient‐derived fibroblasts.

## Discussion

Affected patients had a generalized hyperkinetic movement disorder with chorea and dystonia, which was more prominent during periods of insomnia. Hyperkinetic paroxysmal dyskinesias also occurred during sleep, reminiscent of *ADCY5*‐related disorders.^14^ Hand stereotypies seemed to appear by the age of 3 years in certain patients. A similar evolution from early onset hyperkinetic movement disorder to hand stereotypy has been described in patients with *FOXG1*‐related disease.^15^

There is a selective constraint against missense variants in *NACC1*, making the excess of an identical missense in this gene an extraordinary event. The c.892C>T variant occurs in a CpG dinucleotide within an arginine codon. This CpG pattern is associated with de novo events at numerous loci when advanced paternal age is present.^16^ However, advanced paternal age has not been reported earlier or found in our cohort. These findings are still evocative of a germline recurrent mutational hotspot associated with this neurodevelopmental disorder. To our knowledge, all the cases of *NACC1*‐related disease have been associated with *de novo* mutations.

Mitochondrial dysfunction can cause a wide spectrum of neurological symptoms, including movement disorders (often dystonia in pediatric patients) and epilepsy.^17^, ^18^ The phenotype of the patients with the recurrent *NACC1* missense variant has neurological features, such as dystonia, that may overlap with those observed in primary mitochondrial disorders. In this study, the biochemical results pointing to mitochondrial dysfunction were scarce, showing normal results in blood or plasma lactate and cerebrospinal fluid lactate in all the patients (Data S2). Urine organic acid analyses were normal in other patients except for a slight elevation of lactic acid and citric acid cycle intermediates in patient 3. Mitochondrial function was studied in participant 5 by Schoch et al^9^ (patient 3 in the current study) and analysis of muscle biopsy revealed reduction in several OXPHOS complexes, including complexes I and IV, whereas the evaluation of mitochondrial copy number was normal and mitochondrial DNA genome sequencing did not show any pathogenic variants.^9^ Therefore, we sought to evaluate whether secondary mitochondrial dysfunction is associated with the symptoms observed in our patients. Moreover, the expression of mitochondrial OXPHOS complexes was studied in detail in patient‐derived fibroblasts from patient 1. Our results indicate that in fibroblasts, the *NACC1* p.Arg298Trp variant cell line exhibits over‐expression of complex I in BN gel, but it does not have an immediate effect at the cellular level on the expression of mitochondrial OXPHOS complexes. However, complex I activity was impaired in patient‐derived fibroblasts compared to controls because in‐gel complex I activity when normalized to the level of fully assembled complex I, is decreased in the patient‐derived fibroblasts compared to control activity. Importantly, this finding does not eliminate other possible effects on mitochondrial function and metabolism in different cell types, such as skeletal muscle and neurons. More investigations—for example, by using human induced pluripotent stem cell (iPSC) ‐derived neuronal model systems—are required to better understand the role of NACC1 in mitochondrial dysfunction and in general in this condition.

## Conclusion

We suggest that *NACC1* should be included in the gene panels for hyperkinetic movement disorders and be especially considered in patients with cyclical movement disorders. To understand the pathomechanisms leading to neurological manifestations and to find potential treatment targets to alleviate the symptoms, more cellular studies (preferably using specific cell types, such as neurons and glial cells) and animal models are required.

## Author Roles

(1) Research Project: A. Conception, B. Organization, C. Execution; (2) Statistical Analysis: A. Design, B. Execution, C. Review and Critique; (3) Manuscript Preparation: A. Writing the First Draft, B. Review and Critique.

J.K.E.: 1A, 1B, 1C, 3A

S.M.K.: 1A, 1B, 1C, 3A, 3B

E.L.M.: 1C, 3A, 3B

T.F.: 1C, 3A, 3B

F.S.: 1C, 3A, 3B

B.M.D.: 1C

O.K.: 1A, 3B

M.S.P.:1C, 3B

L.C.:1C, 3B

R.H.:1A, 1B, 3A, 3B

M.K.:1C, 3B

J.U.: 1A, 1B, 1C, 3A, 3B

## Disclosure

**Ethical Compliance Statement:** We hereby confirm that the present study confirms to the ethical standards and guidelines of the journal. We confirm that the ethical principles for medical research involving human subjects (Declaration of Helsinki, WMA, 1975, revised 2000) has been followed. The study was approved by the ethics committee of Oulu University Hospital (POGE, EETMMK 33/2014) and each participating center has followed guidelines and received permission from their local ethics committee The patients have given written and informed consent for online publication of their videos. We confirm that we have read the Journal's position on issues involved in ethical publication and affirm that this work is consistent with those guidelines.

**Funding Sources and Conflict of Interest:** The authors declare that there are no conflicts of interest relevant to this work. J.K.E. has received research grant support from the Alma and K.A. Snellman Foundation, Oulu, Finland; the Arvo ja Lea Ylppö Säätiö, Helsinki, Finland; and the Finnish Cultural Foundation, North Ostrobothnia Regional Fund, Oulu, Finland (grant number, 60152194, 2015). E.L.M. and B.M‐D. have received grant support from the Instituto de Salud Carlos III, Spain, ISCIII (PT20CIII/00009). R.H. has received funding from University of Oulu and Academy of Finland profiling program (grant, 311934). M.A.K. has received funding from the National Institute for Health and Care Research (NIHR) Professorship (NIHR‐RP‐2016‐07‐019), Sir Jules Thorn Award for Biomedical Research (JTA‐017), Rosetrees Trust (CF2\100018), and Medical Research Council (MR/S020136/1). J.U. has received research grant support from the Pediatric Foundation, Finland, the Academy of Finland (Decision number 331 436) and Special State Grants for Health Research in the Clinic for Children and Adolescents, Oulu University Hospital, Finland. F.S has received grant support from, PTC Therapeutics, Astellas Pharmaceuticals, FDA (5R01‐FD005407), and National Institute of Health (5 U54 NS078059 and 5 U54‐NS115198 03). T.F., O.K, M.S‐P., and L.C. did not receive specific funding for this work.

**Financial Disclosures for the Preceding 12 months:** J.K‐E. received a fee from Jazz Pharmaceuticals for joining the TSC Advisory Board meeting and for presenting a lecture. J.U. received a fee from Pfizer for joining Duchenne muscular dystrophy (DMD) Finland Advisory Board meeting. M.A.K. is a founder of Bloomsbury Genetic Therapies and a consultant for this company; she also receives honoraria from PTC.

## Supporting information


**Data S1.** Materials and methods.

**Data S2.** Clinical features and movement analysis of the patients.

**Figure S1.** The brain MRI of patient one with c.892C > T *NACC1* variant.

## References

[1] Sanger TD , Chen D , Fehlings DL , et al. Definition and classification of hyperkinetic movements in childhood. Mov Disord 2010;25(11):1538–1549. 20589866 10.1002/mds.23088 PMC2929378

[2] Kurian MA , Dale RC . Movement disorders presenting in childhood. Continuum (Minneap Minn) 2016;22(4):1159–1185. 27495203 10.1212/CON.0000000000000367

[3] Koene S , Rodenburg RJ , van der Knaap MS , et al. Natural disease course and genotype–phenotype correlations in complex I deficiency caused by nuclear gene defects: what we learned from 130 cases. J Inherit Metab Dis 2012;35(5):737–747. 22644603 10.1007/s10545-012-9492-z PMC3432203

[4] Cordeiro D , Bullivant G , Siriwardena K , Evans A , Kobayashi J , Cohn RD , Mercimek‐Andrews S . Genetic landscape of pediatric movement disorders and management implications. Neurol Genet 2018;4(5):e265. 30283815 10.1212/NXG.0000000000000265 PMC6167181

[5] Carecchio M , Mencacci NE . Emerging monogenic complex hyperkinetic disorders. Curr Neurol Neurosci Rep 2017;17:97. 29086067 10.1007/s11910-017-0806-2 PMC5662693

[6] Neveling K , Feenstra I , Gilissen C , et al. A post‐hoc comparison of the utility of Sanger sequencing and exome sequencing for the diagnosis of heterogeneous diseases. Hum Mutat 2013;34(12):1721–1726. 24123792 10.1002/humu.22450

[7] van Egmond ME , Lugtenberg CHA , Brouwer OF , et al. A post hoc study on gene panel analysis for the diagnosis of dystonia. Mov Disord 2017;32(4):569–575. 28186668 10.1002/mds.26937

[8] Kwong AKY , Tsang MHY , Fung JLF , et al. Exome sequencing in pediatric patients with movement disorders. Orphanet J Rare Dis 2021;16(1):32. 33446253 10.1186/s13023-021-01688-6 PMC7809769

[9] Schoch K , Meng L , Szelinger S , et al. A recurrent de novo variant in NACC1 causes a syndrome characterized by infantile epilepsy, cataracts, and profound developmental delay. Am J Hum Genet 2017;100(2):343–351. 28132692 10.1016/j.ajhg.2016.12.013 PMC5294886

[10] Lyu B , Dong Y , Kang J . A new case of de novo variant c.892C > T (p.Arg298Trp) in NACC1: a first case report from China. Front Pediatr 2021;9:754261. 34869110 10.3389/fped.2021.754261 PMC8634650

[11] Kim MJ , Yum MS , Seo GH , Ko TS , Lee BH . Phenotypic and genetic complexity in pediatric movement disorders. Front Genet 2022;13:829558. 35719373 10.3389/fgene.2022.829558 PMC9198294

[12] Korutla L , Wang P , Jackson TG , Mackler SA . NAC1, a POZ/BTB protein that functions as a corepressor. Neurochem Int 2009;54(3–4):245–252. 19121354 10.1016/j.neuint.2008.12.008

[13] Xie Q , Tong C , Xiong X . An overview of the co‐transcription factor NACC1: beyond its pro‐tumor effects. Life Sci 2024;336:122314. 38030057 10.1016/j.lfs.2023.122314

[14] Chang FCF , Westenberger A , Dale RC , et al. Phenotypic insights into ADCY5‐associated disease. Mov Disord 2016;31(7):1033–1040. 27061943 10.1002/mds.26598 PMC4950003

[15] Wong LC , Wu YT , Hsu CJ , Weng WC , Tsai WC , Lee WT . Cognition and evolution of movement disorders of FOXG1‐related syndrome. Front Neurol 2019;10:641. 31316448 10.3389/fneur.2019.00641 PMC6611493

[16] Bernhardt L , Dittrich M , Prell A , et al. Age‐related methylation changes in the human sperm epigenome. Aging (Albany NY) 2023;15(5):1257–1278. 36849136 10.18632/aging.204546 PMC10042684

[17] Musumeci O , Oteri R , Toscano A . Spectrum of movement disorders in mitochondrial diseases. J Transl Genet Genom 2020;4:221–237.

[18] Bindoff LA , Engelsen BA . Mitochondrial diseases and epilepsy. Epilepsia. 2012;53(Suppl. 4):92–97. 22946726 10.1111/j.1528-1167.2012.03618.x

[19] López‐Martín E , Martínez‐Delgado B , Bermejo‐Sánchez E , Alonso J . SpainUDP: the Spanish undiagnosed rare diseases program. Int J Environ Res Public Health 2018;15(8):1746. 30110963 10.3390/ijerph15081746 PMC6121381

