# Supplementary material for: Hyperkinetic Movement Disorder Caused by the Recurrent c.892C>T NACC1 Variant
Source: Mov Disord Clin Pract. 2024 May 2;11(6):708–15. doi: 10.1002/mdc3.14051 (PMC11145100; doi:10.1002/mdc3.14051)
Supplement: Supplementary file 1 — Data S1. Materials and methods. [file MDC3-11-708-s002.docx]

**Supplement 1: Materials and methods**

**Materials and methods**

**Cell culture**

Primary patient-derived dermal fibroblasts with heterozygous *De Novo* NACC1 pArg298Trp variant and normal human dermal fibroblasts (NHDF, Promocell, Heidelberg, Germany) as controls were cultured in a fibroblast growth medium (Dulbecco’s Modified Eagle’s Medium (DMEM; Corning, Manassas, VA, USA) supplemented with 10% fetal bovine serum (FBS Good; Pan Biotech, Aidenbach, Bavaria, Germany), 1% L-glutamate (Gibco, Thermo Fisher Scientific, Hampton, New Hampshire, United States), and 1% penicillin-streptomycin (GE Healthcare HyClone™, Fisher Scientific).

**RT-PCR**

RT-PCR was used to study the expression of the *NACC1* c.892C>T variant in patient-derived fibroblasts. RNA extraction was performed from cultured fibroblasts with a RNeasy Plus Mini Kit (Qiagen, Hilden, Germany). RT-PCR was performed by using QIAGEN OneStep RT-PCR Kit (Qiagen, Hilden, Germany) and primer pair with forward primer: CGCAAGATGGCCAAGTTCTC and reverse primer: GCGGTTCCCAATCTGGTTGA. After the RT-PCR, the cDNA products were run on 1.5% Agarose gel and 462 nt bands were cut out from the gel. The PCR products were purified from the gel with NucleoSpin Gel and PCR Clean‑up Mini kit for gel extraction and PCR clean up (Macherey-Nagel). The purified cDNA was used for Sanger sequencing at Biocenter Oulu Sequencing Center (University of Oulu, Finland) using the same primers as those used for the RT-PCR above.

**Quantitative PCR**

Quantitative PCR was used to examine the expression level of NACC1 in the patient-derived fibroblasts. RNA extraction was performed from cultured fibroblasts with a RNeasy Plus Mini Kit (Qiagen, Hilden, Germany). Reverse transcription of the mRNA to cDNA was done by using QuantiTect Reverse Transcription kit (Qiagen, Hilden, Germany) according to the manufacturer’s instructions. qPCR was performed according to the manufacturer’s instructions (IQTM SYBR Green Supermix, Bio-Rad, Hercules, CA, USA) by CFX ConnectTM Real-Time System (Bio-Rad, Hercules, CA, USA). Tm of the analysis was adjusted at 60°C. The TATA-box binding protein (TBP), ribosomal protein L13a (RPL13A) and Transferrin receptor (TFRC) were used as endogenous reference genes. The primers were designed using NCBI primer blast and the used qPCR oligos are presented in Table 1.

Table 1. Primers sequences for qPCR

| **Target name** | **Primer name** | **Primer sequence** | **Product length** |
| --- | --- | --- | --- |
| TBP | hTBPq_F | CCACTCACAGACTCTCACAAC | 127 |
|  | hTBPq_R | CTGCGGTACAATCCCAGAACT |  |
| RPL13A | hRPL13Aq_F | CGGACCGTGCGAGGTAT | 114 |
|  | hRPL13Aq_R | CACCATCCGCTTTTTCTTGTC |  |
| TFRC | hTFRCq_F | TCTAACTTGTTTGGTGGAGAACC | 83 |
|  | hTFRCq_R | TCCACATGACTGTTATCGCCA |  |
| NACC1 | hNacc1q_F | CTTCTTTGACCGGAACACGC | 113 |
|  | hNacc1q_R | AGTACTTGACAGCGTGGAGC |  |

**Immunoblotting**

Whole-cell protein extracts were prepared in 1.5% n-Dodecyl ß-D-maltoside (Sigma-Aldrich, St. Louis, MO, USA) in phosphate-buffered saline (PBS, Gibco, Thermo Fisher Scientific) containing protease inhibitor cocktail (Pierce™, Thermo Fisher Scientific, Rockford, IL, USA). Twenty micrograms of protein per sample was loaded onto 4–20% Mini-PROTEAN® TGX™ Precast Protein Gel (Bio-Rad, Hercules, CA, USA). Western blotting was performed with Trans-Blot® Turbo™ Transfer System (Bio-Rad) onto a Trans-Blot Turbo Mini Nitrocellulose membrane (Bio-Rad). Unspecific binding was blocked with 5% non-fat dry milk (Valio, Finland) in Tris buffered saline (Medicago, Uppsala, Sweden) with 0.01% tween 20 (Fisher Scientific, Geel, Belgium). Protein detection was done with standard protocol using the primary antibodies rabbit anti-NACC1 (ab29047, Abcam, Cambridge, United Kingdom; 1:250) and rabbit anti-GAPDH (GTX100118, Genetex, Irvine, CA, USA; 1:10 000). Secondary antibody used was Goat Anti-Rabbit IgG H&L horseradish peroxidase (Abcam, ab97080, 1:10 000). The bands were detected using Westernbright^TM^ ECL reagent (K-12045-D20, Advansta, Menlo Park, CA, USA) and LAS-3000 Imager Analyzer system (Fujifilm, Tokyo, Japan).

Signal intensities for protein quantification from the blots were measured using Image J software Fiji (NIH, Bethesda, MA, USA [1]. P-values were calculated using two-tailed student’s two-sample T-test assuming equal variances.

**Immunocytochemistry**

Briefly, fibroblasts grown on glass coverslips were fixed in 4% paraformaldehyde (PFA, Pierce™, Thermo Fisher Scientific) in PBS and permeabilized with 0.1% Triton X‐100 (Sigma-Aldrich). Unspecific binding was blocked with 5% bovine serum albumin (BSA, Fisher Bioreagents™) in PBS, followed by incubation with primary antibodies rabbit anti-NACC1 (ab29047, Abcam; 1:250), rabbit anti-Tom20 (#42406, Cell Signaling Technology, Danvers, MA, USA; 1:200), and Phalloidin:FITC (PF7501, ECM Biosciences, Versailles, KY, USA; 1:500). Secondary antibody was Alexa Fluor546 goat anti-RABBIT IgG (A11010, Invitrogen, Thermo Fisher Scientific; 1:2000). Nuclei were stained using Hoechst (Invitrogen, H3570, 1:10000). In addition, coverslips were mounted onto slides using fluorescence mounting medium (Dako, Santa Clara, California, United States).

Images were captured with Zeiss Axio Imager.D2 using 63x Oil objective and Zen (blue edition) software.

**Blue native PAGE**

Blue native polyacrylamide gel electrophoresis (BN-PAGE) was performed to compare expression level and assembly of mitochondrial complexes in the cultured patient-derived and control fibroblasts. BN-PAGE was carried out according to protocols, as previously described [2,3].

The proteins were blotted on nitrocellulose membrane by semi-dry protein transfer. The mitochondrial complexes were probed with the following primary antibodies: complex I/NDUFA9 (#A21344 Molecular Probes, Life Technologies, Carlsbad, CA, USA); complex II/SDHA (#ab14715, Abcam); complex III/UQCRC2 (#ab14745 Abcam); complex IV/COXIV (#ab14744 Abcam) and complex V/ATP5A (#ab14748 Abcam).

HRP-conjugated secondary antibodies Goat Anti-Rabbit IgG H&L horseradish peroxidase (#ab97080, Abcam 1:10,000) and Goat Anti-Mouse IgG H&L horseradish peroxidase (ab6789, Abcam, 1:10,000) were used for detection. Protein bands were detected by WesternBright ECL Spray (Advansta, CA, U.S.A.). LAS-3000 Luminescent Image Analyzer (Fuji Photo Film, Tokyo, Japan) was used for chemiluminescence signal detection. Signal intensities for protein quantification from the blots was measured with Image J software Fiji (NIH, Bethesda, MA, USA [1]. P-values were calculated using two-tailed student’s two-sample T-test assuming equal variances.

**In-gel activity assay**

The activity of mitochondrial complex I was studied using in-gel activity assay according to protocol by Nijtmans and others [2]. First, the samples were run on BN-PAGE 6-15% gradient gel, with 4% stacking gel as described above. The activity of complex I was assessed by in-gel color reaction caused by the oxidation of electron donor diamino-NADH and reduction of nitrotetrazolium blue chloride catalyzed by the dehydrogenase subunit of complex I.

**References**

[1] Schindelin J, Arganda-Carreras I, Frise E, et al. Fiji: an open-source platform for biological-image analysis. *Nat. Methods* 2012; 9(7): 676-682.

[2] Nijtmans LGJ, Henderson NS, Holt IJ. Blue Native electrophoresis to study mitochondrial and other protein complexes. *Methods* 2002; 26(4): 327-334.

[3] Ugalde C, Janssen RJRJ, van den Heuvel LP, et al. Differences in assembly or stability of complex I and other mitochondrial OXPHOS complexes in inherited complex I deficiency. *Hum Mol Genet* 2004; 13(6): 659-667.
