# Supplementary material for: Hyperkinetic Movement Disorder Caused by the Recurrent c.892C>T NACC1 Variant
Source: Mov Disord Clin Pract. 2024 May 2;11(6):708–15. doi: 10.1002/mdc3.14051 (PMC11145100; doi:10.1002/mdc3.14051)
Supplement: Supplementary file 2 — Data S2. Clinical features and movement analysis of the patients. [file MDC3-11-708-s001.docx]

**Supplement 2: Clinical features and movement analysis of the patients**

**Clinical features**

Patient one

Patient one is a seven-year-old boy, who was referred for further investigations due to an alternating squint, poor eye contact, and increased muscular tone at the age of four months. He initially presented cyclic dysautonomia symptoms: sinus tachycardia, vomiting, fever, sweating, diminished urine secretion and urinary retention, constipation, and frequent loose stools. He had periods of insomnia that alternated with periods of sleepiness in two-week cycles. Until two years of age, he cried inconsolably during insomnia cycles. At the age of one year, sodium valproate treatment was initiated due to a prolonged focal onset seizure. Epilepsy was stabilized with gabapentin treatment, and atenolol was effective to treat sinus tachycardia. The patient has profound intellectual disability, axial hypotonia, postnatal microcephaly (head circumference 45.8 cm/-3.9 standard deviation (SD) at two years of age), bilateral cataracts, reflux, eating difficulties and malnutrition requiring jejunostomy feeding.

Brain MRIs revealed a thin corpus callosum, hypomyelination, and cerebral atrophy, which did not progress during the seven-year follow-up (Supplementary Figure 1A-F). Interictal EEG revealed a mild background abnormality, a high occipital 2–3 Hz delta (particularly during sleep), and no epileptiform discharges. Video EEG under gabapentin medication was normal in wakefulness and sleep at the age of three years. There were no epileptic findings in EEG during hyperkinetic movements, which also occurred during sleep.

Biochemical investigations were normal; blood lactate 1.21-1.23 mmol/l (0.33-1.33), CSF lactate 1.52 mmol/l (1.1-2.2), and urine organic acids normal. Reanalysis of WES (whole-exome sequencing) was done to search for *NACC1* after clinical geneticist had discovered Scholl et at article while making searches with patient´s clinical features and *N*ACC*1* missense c.892C>T (p.Arg298Trp) was found.

Movement disorder (Video S1) was recognized in the form of involuntary tongue protrusion at the age of six months. Generalized hyperkinetic movement disorder with chorea and dystonia was evident after one year of age. The patient experienced myoclonic jerks during both sleep and wakefulness. The hyperkinetic movement disorder was more prominent during periods of insomnia, which alternated with periods of sleepiness in two-week cycles. Muscle tone increased during hyperkinesia. The patient had arousals from sleep due to episodic generalized hyperkinetic movements. Hand mouthing and biting during the hyperactive stage were initially present until two years of age, then stopped but began again with increasing intensity at the age of five years. Clonidine was initiated at the age of three years and was partially helpful for insomnia, vomiting, and tachycardia during the hyperactive stage. No objective benefits for the movement disorder were observed with the use of gabapentin, L-dopa , nitrazepam, diazepam, or lorazepam. Clonazepam has usually helped with the hyperkinetic movement disorder and partially with muscle hypertonia.

Patient two

Patient two is a nine-year-old male presenting with apnea crises secondary to gastroesophageal reflux, swallowing difficulties, and choking at the age of three months. He manifested with cyclic dysautonomia symptoms (vomiting, hypersalivation, constipation, and urinary retention), irritability, and crying. He had periods of insomnia that alternated with periods of sleepiness in 10–15-day cycles. Infantile spasms were diagnosed, and ACTH treatment was initiated at the age of six months. After the treatment with ACTH, EEGs normalized. The patient has profound intellectual disability, no expressive language, bilateral cataracts, postnatal microcephaly, global hypotonia, joint hypermobility, and required surgery for gastroesophageal reflux with partial effect and found to have esophageal spasms in micromanometry.

Brain MRIs at the age of six and eighteen months were normal. EEG showed hypsarrhythmia. Biochemical investigations were normal; plasma lactate 15 mg/dl (normal values: 5.7-22), CSF lactate and CSF organic acids normal.

The *NACC1* missense c.892C>T (p.Arg298Trp) variant was identified after trio-WES. Subsequent phenotype-genotype correlation led to establish this variant as causative.

Movement disorder was first recognized in the form of minor myoclonic jerks during sleep at the age of nine months. Subsequently, the myoclonic jerks became more evident and appeared when the child was awake. Choreic tremulous jerks during sleep were noticed at the age of two years. Dystonic crises with an opisthotonic posture, hyperextension of limbs, and upward eye deviation during periods of insomnia were evident at the age of four years. Moreover, increased muscle tone of the limbs was noticed during the dystonic episodes. Stereotypic hand mouthing was more prominent during cyclic insomnia. Clonidine treatment —initiated at five years of age— was effective for dystonic crises and spasticity but had no effect on choreic tremulous jerks during sleep. There was no benefit to the anxiety or sleep from levomepromazine or diazepam. Nifedipine was helpful for esophageal spasms and nauseas.

Patient three

Patient three is a 15-year-old girl, individual 5 described by Schoch et al. (2017), who was diagnosed with reflex myoclonic epilepsy of infancy at the age of five months.^1^ She subsequently manifested with infantile spasms with hypsarrhythmia and drug-resistant epilepsy, hypotonia, cyclic dysautonomia, and inconsolable crying. She had profound intellectual disability, feeding difficulties requiring gastrojejunal tube feeding, bilateral cataracts, microcephaly, precocious puberty, and severe iron-deficiency anemia.

Brain MRI revealed delayed brain myelination at the age of five months. Muscle biopsy showed reduction in several respiratory chain complexes, including complexes and I and IV. Mitochondrial copy number was normal in the muscle sample and sequencing of the mitochondrial genome was normal.^1^

EEG was recorded during the dyskinetic movements with no epileptiform activity. Biochemical investigations showed normal venous plasma lactate ranging from 0.7-1.6 mmol/liter (normal reference range: 0.7-1.6). In plasma amino acids alanine was elevated; 511 micromoles per liter (normal reference range: 148-420). In urine organic acid analysis (qualitative organic acid analysis) there was slight elevation of lactic acid and citric acid cycle intermediates: citric, aconitic, succinic, and 2-oxoglutaric.

The *NACC1* missense c.892C>T (p.Arg298Trp) variant was identified from clinical exome data in Baylor Genetics as a part of the research project from Schoch et al (patient 5).

Movement disorder was evident from the age of one year in the form of multifocal myoclonus, perioral dyskinesia, generalized choreoathetoid movements, and dystonia. Hyperkinetic movement disorder was more prominent during irritability and insomnia periods. Intensity of the hyperkinetic movements during irritability and insomnia periods reduced with age. Stereotypical hand clasping in midline, hand mouthing, and biting was present by three years of age. Generalized hyperkinetic movements appear during sleep. Spasticity has increased with age.

Patient four

Patient four is a 16-year-old boy, who first presented with four failed hearing exams, significant colic/temperament difficulty, presumed gastroesophageal reflux, feeding difficulties, and variable esotropia. He was treated for infantile spasms with hypsarrhythmia, with no benefit from ACTH injections, topiramate, zonisamide, and vitamin B6 at the age of four months. He manifested with significant cyclical episodes of inconsolable irritability and insomnia lasting two to five days and occurring every seven to ten days. Before the episodes he had increased spasticity, insomnia, inappropriate laughter, and nonverbal vocalization. He had a four-month seizure free period with the use of diazepam and zonisamide, followed by a relapse of cryptogenic infantile spasms with a second round of ACTH treatment. This was followed by treatment with lamotrigine, phenobarbital, and levetiracetam.

He has microcephaly, profound intellectual disability, failure to thrive with gastrostomy tube placement, hypotonia, bilateral cataracts, and periods of hypersalivation with excessive mucous production, vomiting, sweating, constipation, urinary retention, and flushed, cool clammy hands and feet, small umbilical hernia, bilateral cryptorchidism, cortical visual impairment, and mild low-tone and moderate high-tone sensorineural hearing loss.

Brain MRIs showed decreased white matter at seven months of age, with diffuse cerebral atrophy and delayed myelination at both one and three years of age. Interval resolution of previously seen delayed myelination and continued diffuse atrophy of both cerebral hemispheres and, to a lesser extent, the cerebellar hemispheres were noticed at the age of 12 years. MRI spectroscopy showed no significant abnormalities at the age of seven months.

Interictal EEG showed diffuse slowing and poor organization of the background. 1 Hz flash provoked spikes that were associated with body jerks at the age of eight years and there was generalized suppression in the background.

Biochemical investigations showed normal results. CSF pyruvate 1.0 (0.54-1.7mg/dl), CSF lactate 1.3 (0.8-2.4 mmol/L), serum lactate <9.0mg/dl, in urine organic acid no unusual organic acids.

Mitochondrial respiratory chain enzymatic analysis was performed on skeletal muscle. Citrate synthase activity was increased, thereby suggesting mitochondrial proliferation. The activities of several respiratory chain complexes were reduced, with a more notable deficiency of complex I activity.

WES sent in 2020 identified the *NACC1* missense c.892C>T (p.Arg298Trp) variant thought to be causative from the beginning.

Movement disorder first presented with fisting and tongue protrusion by two months of age. He developed axial hypotonia with increased tone in arms and legs, consistent with a mild to moderate quadriparesis by nine months of age. Further, sporadic episodes characterized by extensor arching and a startle-like movement and myoclonic jerks when falling asleep which did not correlate with EEG abnormalities were observed at one year of age. Jerks also occurred upon waking. Opisthotonic posturing when irritable with cries were reported by two years of age. Choreic movements of the body with increased tone with flexor posturing at his elbows and knees as well as stereotypical hand clasping and hand mouthing were observed by three years of age. During irritable episodes with insomnia, increased spasticity, teeth grinding and thumb biting were noticed. Generalized hyperkinetic movements appeared during sleep. Spasticity is reported to have increased with age.

Multiple medications were attempted, with no significant benefit for cyclic irritability, including gabapentin, nortriptyline, hydrocodone, tramadol, cyproheptadine, chloral hydrate, baclofen, morphine, clonazepam, quetiapine, prednisone, diazepam, and guanfacine. Clonidine and trazodone were helpful in promoting rest. The treatment with tetrahydrocannabinol and onabotulinum toxinA/phenol injections were most helpful for treating spasticity and involuntary movements.

**Reference:**

1. Schoch K, Meng L, Szelinger S, et al. A recurrent de novo variant in NACC1 causes a syndrome characterized by infantile epilepsy, cataracts, and profound developmental delay. Am J Hum Genet 2017: 100(2): 343-351.

**Supplementary figure 1:**

Midsagittal T1-weighted brain MRI showed thin corpus callosum at the age of five months, without high-signal intensity of the splenium of the corpus callosum (A, arrow) due to delayed myelination. At the age of 17 months, the signal of the corpus callosum (B, arrow) had increased, thereby suggesting progression of the myelination; however, there was still thinning of the corpus callosum. At the age of seven years, the size of the corpus callosum did not increase, thereby suggesting hypoplasia (C, arrow). The forebrain was small compared to the hindbrain and face, thereby suggesting microcephaly (C).

Axial T2-weighted brain MRI at the age of five months demonstrated hypointense myelin in the posterior limbs (arrows) but not in the anterior limbs (arrowheads) of the internal capsules, thereby suggesting delayed myelination (D). At the age of 17 months, myelination had progressed, revealing myelin in the corpus callosum (arrows) and anterior limbs of the internal capsules (arrowheads), but the myelination was still delayed (E). At seven years of age, the maturation of the subcortical white matter (F, arrows) remained unmyelinated, thereby suggesting permanent hypomyelination. Brain MRI revealed a decreased volume of the cerebral white matter, thereby suggesting diffuse cerebral atrophy that did not progress during the seven-year follow-up (D-F).

**Movement analysis**

**Patient one** (Video S1)

Age: One year and one month

The child is lying on a mat. There is a tendency for the mouth to be held open. Orolingual dyskinesia is evident with repeated tongue protrusion and drooling. There are also subtle generalized choreiform movements of the limbs. He is able to fix and follow, but there is brief evidence of a convergent squint on right. On attempting to reach for the toy, dystonic posturing is evident in the upper limbs, with dystonic finger postures. Occasionally, there is bilateral hand fisting. On lifting to the upright position, there is evidence of axial hypotonia and head lag.

Age: One year and two months

The child is being supported in the sitting position. Repeated tongue protrusion with mouth opening is evident. There is axial hypotonia and reduced head control. There is intermittent striatal toe bilaterally, with foot clawing. Hands are also fisted bilaterally. Brief lower limb scissoring is seen on vertical suspension. Occasionally, both upper limbs show an extended and pronated posture with fisting of the hands.

Age: One year six months

The child is lying down. The mouth remains open most of the time with subtle orolingual dyskinesia. Occasional vocalizations are audible. Throughout the video, he appears to have stiff limbs. Scissoring is evident as well as periods where the legs remain fully extended, with foot clawing. He is able to repeatedly roll from his back to the right side by lifting his legs and rolling over. Hand fisting and intermittent dystonic posturing of the fingers is also seen.

Age: Two years four months

The child is lying in bed. Tongue protrusion is evident. Some movements of the upper and lower limbs are noted and they appear to be mostly voluntary.

Age: Two years and four months

The child is initially lying on a mat. There are repeated episodes of tongue protrusion and mouthing. Subtle upper limb posturing is noted. He is able to roll from back to side. Voluntary movements of the upper limbs are seen, that occasionally have a jerky, dyskinetic quality. Marked axial hypotonia and head lag is present when pulling to sit and when sitting. There is also significant truncal instability on sitting.

Age: Two years and five months

The child is lying on a mat. There is orolingual dyskinesia, as previously seen. Striatal toe is frequently seen bilaterally. One short startle was noted at the beginning of the video. Movements of all four limbs appear mainly voluntary (or an attempt at being purposeful), but both upper and lower limb postures are seen during these movements.

Age: Three years

The child appears to be asleep for the duration of the video. During sleep, there appear to be episodic generalized jerky and low to moderate amplitude choreiform movements, with a few possibly tremulous movements predominantly of the upper limbs, but also affecting the lower limbs. The episodes are short and appear to last for <30 seconds. Occasionally, there is either fisting or dystonic posturing of the right hand.

**Patient two** (Video S2)

Age: One year and four months

The child is in distress, crying for the duration of the video. There is evidence of retching in the beginning the video. It is difficult to determine exactly, but there is the suggestion from the video that when the child is held more prone, there are a few possibly choreiform movements of the upper limbs.

Age: Four years eight months

The child is seated with support on a lap. The right arm is repeatedly lifted and held in what appears to be a dystonic posture, arm extended, wrist flexion. On one occasion, the right arm is held extended, with wrist flexion and also pronation. Then, the entire body appears to be in an opisthotonic posture, with retrocollic neck posture, truncal arching, and extension of the legs and bilateral equinus posturing of the feet.

Age: Four years and eight months

The child is on a lap and appears to be in distress. There appear to be two episodes of generalized dystonic posturing, each lasting for a duration of less than one minute. During the episode, there is mouth opening, whole body arching, and what appears to be retrocollis, with dystonic posturing of the upper limbs and possible dystonic tremor evident in the hands. Feet also show dystonic postures with intermittent clawing and also striatal toe.

Age: Six years and six months

The child is lying on a bed, awake. For a brief period, there is tongue protrusion at the beginning of the video. He has a tendency to bring his hands to his mouth. There are a few low amplitude choreiform movements affecting the right hand, with a degree of hand fisting. More subtle choreiform movements are also evident in the left hand. There is frequent elevation of both legs and possibly similar, rather subtle, choreiform movements distally in the lower limbs.

Age: Six years and six months

The child appears asleep in bed. A generalized jerky movement is seen at the beginning of the video. A few subtle hyperkinetic movements of the fingers are also evident.

Age: Six years and six months

The child is lying in a chair. There is an episode of leg elevation associated with possibly striatal toe on the left and movements of the arms that appear somewhat dyskinetic/choreiform.

Age: Six years and six months

The child is lying face down in the bed. When turning, there are a few choreiform movements, which are most evident in the hands and feet.

Age: Six years and six months

The child is lying in the bed. On turning, low amplitude choreiform movements are evident in the limbs. When lying prone, his legs adopt a posture with flexed knees, which is associated with choreiform movements of the feet and some foot clawing. Possible striatal toe on the left foot is evident. The mouth is held open during some parts of this episode.

Age: Six years and six months

The child is lying on a bed. Both hands are intermittently brought to the mouth. Movements are very similar to those described previously—generalized low-amplitude choreiform movements, particularly distally, affecting the hands and feet, periodic open mouth, and possible upper and lower limb dystonic posturing.

Age: Six years and six months

The child is asleep on a bed. Movements as previously described — generalized low-amplitude choreiform movements, particularly distally, affecting the hands and feet, periodic open mouth, and possible upper and lower limb dystonic posturing—are seen, evident on turning.

Age: Six years and six months

The child is seated. Initially there is tongue protrusion. There are repeated movements of the head from left to right.

Age: Six years and six months

The child is lying prone and his legs and trunk appear rather still, initially. The fingers of the left hand are overlapping and held in a slightly fisted position. The right arm is extended. Subtle dyskinetic/choreiform movements of both hands are frequently seen. There is intermittent tongue protrusion. Over time, he pushes up from prone position with bilateral hand fisting and flexion at the knees, with crossing of the legs.

**Patient three**

Age: One year and four months

The child is lying with the right arm flexed at the elbow and the left arm pronated and extended. Early movements are reminiscent of spasms, but there is also possibly a few multifocal myoclonus and generalized jerky dyskinetic movements.

Age: One year and eight months

The child appears to be lying on a cot. There is evidence of perioral dyskinesia. The left arm is held with flexion at the elbow and slightly fisted at the wrist. There are possibly a few athetoid movements of the trunk and choreiform/hyperkinetic movements of the arms as well. Occasionally, both arms extend with fisted hands.

Age: Two years and five months

The child is lying on a mat. There is perioral dyskinesia. The arms are held in flexion at the elbow bilaterally, and there is intermittent posturing of the upper limbs and both hands. At 30 seconds, there is evidence of a spasm. There is intermittent foot clawing. Brief jerky, possibly myoclonic, movements are seen in the upper and lower limbs. At 53 seconds, she has an event that is characterized by tonic extension of the body and right arm, associated with some lower limb (possibly myoclonic) jerky movements.

Age: Two years and eight months

The child is lying on a mat. She has what appears to be involuntary hyperkinetic movements, mainly of her upper limbs but also affecting her lower limbs. Movements appear choreiform and occasionally almost ballistic in nature.

Age: Two years and ten months

There are two videos—in one the child is lying down and the other the child is sitting. Both show her having generalized choreoathetosis with orolingual dyskinesia.

Age: Two years and ten months

There are two videos with the child lying down, showing generalized dyskinetic movements, with choreoathetosis.

Age: Three years and one month

The patient is seated in a chair and smiling periodically, with a few more subtle hyperkinetic movements of the hands.

Age: Three years and four months

The child is lying on a mat, with generalized choreoathetosis of the limbs and trunk as well as perioral dyskinesia.

Age: Three years and ten months

The child is lying on a cot and is being shown toys. She smiles periodically and there is evidence of orolingual dyskinesia and a few involuntary upper limb movements.

Age: Four years and one month

The child is lying in semi-prone/prone position. She brings her hands together repeatedly with a few, possibly stereotypical, dyskinetic hand movements. There is evidence of orolingual dyskinesia.

Age: Five years

The child is seated in a chair and appears to be in discomfort. There is evidence of generalized choreoathetosis affecting the trunk and limbs, with intermittent mouth opening.

Age: Seven years and six months

The child is seated in a chair and appears to be uncomfortable. Her head is dropping forward. She has intermittent hyperkinetic movements of her arms and legs and occasionally brings her knees to her chest.

Age: Nine years and four months.

The child is lying on a mat. The hands are repeatedly clasped in the midline and brought to the mouth. There are generalized hyperkinetic/dyskinetic movements of the upper/lower limbs as well as perioral dyskinesia, which is more evident when she lifts her head.

Age: Nine years and four months

The child is seated in a chair. The hands are repeatedly brought to the midline clasping position and also to the mouth, which is often held open. There are intermittent hyperkinetic movements of the head, trunk, and all four limbs.

Age: 12 years

The child is lying on a bed. There is evidence of mild generalized dyskinetic movements affecting the limbs and trunk and a few movements indicating subtle orolingual dyskinesia, with tongue protrusion. The hands are occasionally brought to the midline and clasped.

**Patient four** (Video S3)

Age: 14 years

Section 1: The child is seen sitting in a wheelchair during clinical examination. His right leg is crossed over his left and he holds this foot with his hands. These upper limb voluntary movements appear jerky and dyskinetic and a few subtle hand and finger posturing is also noted. Both feet show clawing postures. There is also intermittent tongue protrusion and orolingual dyskinesia.

Section 2: When lying down, his initial position shows both knees and elbows flexed. When he attempts to roll, the upper limbs again look dyskinetic and there is evidence of orolingual dyskinesia and intermittent tongue protrusion. Foot clawing is also evident. When prone, he is able to lift his head and his knees/elbows are again flexed.
