# Supplementary figures and images for: Hyperkinetic Movement Disorder Caused by the Recurrent c.892C>T NACC1 Variant
Source: Mov Disord Clin Pract. 2024 May 2;11(6):708–15. doi: 10.1002/mdc3.14051 (PMC11145100; doi:10.1002/mdc3.14051)

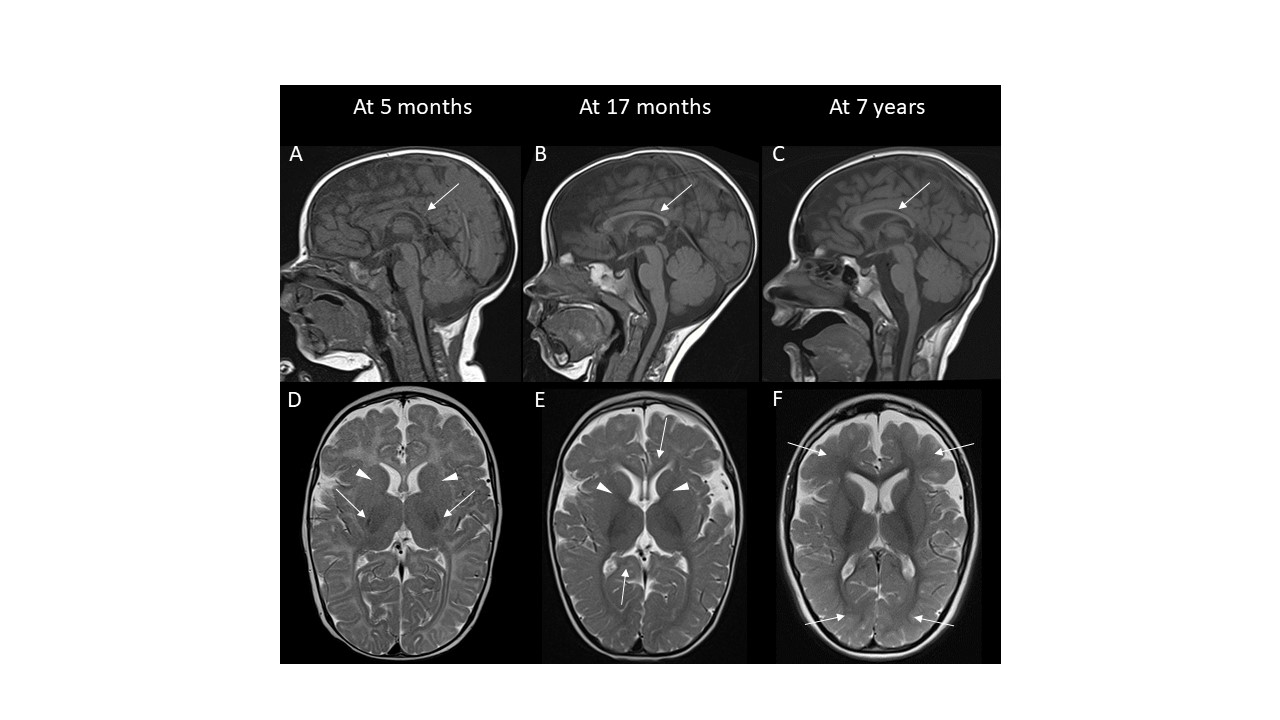

Supplement: Supplementary file 3 — Figure S1. The brain MRI of patient one with c.892C > T NACC1 variant. [file MDC3-11-708-s003.jpg]
